# Tiny Guardian—Monitoring Antimicrobial Childhood Exposure in Low-Resource Settings

**DOI:** 10.3389/ijph.2024.1607146

**Published:** 2024-04-04

**Authors:** Flavia Rosa-Mangeret, Damilola Oladapo, Emely Maria Shefa, Noemie Wagner, Riccardo E. Pfister

**Affiliations:** ^1^ Division of Neonatal and Pediatric Intensive Care, Geneva University Hospitals, Geneva, Switzerland; ^2^ Faculty of Medicine, University of Geneva, Geneva, Switzerland; ^3^ School of Natural and Environmental Sciences, Newcastle University, Newcastle upon Tyne, United Kingdom; ^4^ Faculty of Health and Medical Sciences, Copenhagen, Denmark; ^5^ Division of Pediatric Infectious Diseases, Geneva University Hospitals, Geneva, Switzerland

**Keywords:** antimicrobialresistance, childhealth, globalhealth, antimicrobialstewardship, digitalhealth

## Problem Statement

The escalating threat of antimicrobial resistance (AMR) is leading to a global health crisis, particularly pronounced in Sub-Saharan Africa and Southeast Asia, where alarming AMR-related mortality rates underscore the gravity of the issue [1, 2].

Children aged 0–5 years carry an undue burden of inappropriate antibiotic prescriptions, a challenge brought on by limited access to diagnostic tools and healthcare infrastructure. For example, up to 80% of children with upper respiratory illnesses may receive antibiotic treatment, despite a significant proportion of these infections being viral [3]. The high prevalence of antibiotic administration persists in the absence of robust antimicrobial stewardship programs and a misconception about the harmlessness of antibiotics.

Beyond AMR, the consequences of antibiotic overuse extend to fundamental health aspects of young children, such as disruption of the microbiome, which might lead to compromised growth and increased susceptibility to chronic diseases such as diabetes, obesity, inflammatory bowel disease, asthma, and allergies cast a long-lasting shadow over the wellbeing of young individuals [4, 5].

The lack of antibiotic exposure quality data may lead to recurrent and prolonged use of antibiotics by changing healthcare actors, especially during childhood when viral infection is common. The absence of population-wide antibiotic usage data hinders knowledge-based targeting for healthcare policies and interventions. Effectively tracking individual antibiotic usage from birth to 5 years would provide essential insights for clinical practice, policy formulations, and patient empowerment, especially in low- and middle-income countries.

## Methodology

This project, developed at the Sustainable Development Goals (SDG) Summer School in July 2023 and organised by the University of Geneva, brought together undergraduate students (ES, DO) to address global health challenges on the African continent. Guided by experts (NW, REP) and a PhD coach (FRM), the team employed open science and rapid prototyping to select USSD for tracking antibiotic use. USSD, or ‘Unstructured Supplementary Service Data’, is a real-time communication platform used on any type of mobile phones. Its simplicity and reach have proven effective in Sub-Saharan Africa for healthcare information dissemination and data quality improvement, showcased in various studies, especially in resource-constrained, remote areas and even on basic phones [6–9]. With cell phone network coverage at 87% in Africa in 2022 but only 45% internet access, USSD is ideal, even more so with its offline capabilities [10].

## Introducing *Tiny Guardian*

*Tiny Guardian* represents a universally accessible, USSD-based antibiotic usage platform. Through a series of USSD prompts and responses, users can input data, receive information, and interact with the platform.

## How Does *Tiny Guardian Work*?

**FIGURE 1:**
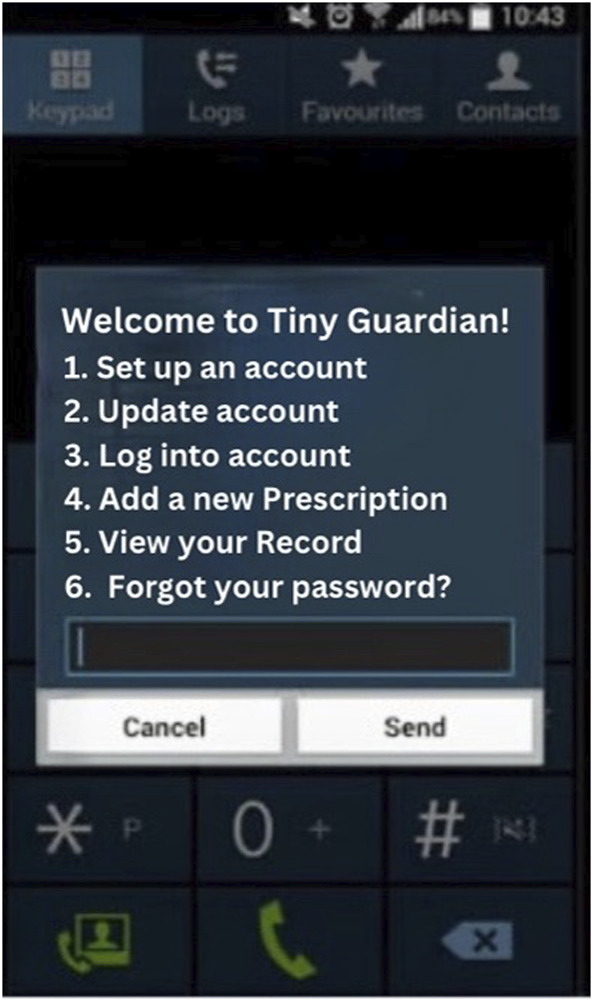
Tiny Guardian Welcome page—smart phone interface (Geneva, Switzerland, 2023).

When a child is born or a caregiver consults a doctor or health practitioner, they are informed about the *Tiny Guardian* and given the general USSD dial number. The caregiver can then generate the initiating USSD code. The platform triggers interactions attributed to each unique identifier (Figure 1).

With the individual child’s USSD PIN, initiating code, and caregivers’ consent, healthcare prescribers can read past antibiotic history and trigger a new USSD ‘conversation’. Whenever deciding on a new prescription, the health practitioner is allowed to input information with a personal USSD code.

The *Tiny Guardian* only collects minimal patient information, that is, age (in years and months, without days), introduced once and updated automatically at new prescription, route of administration (intravenous or oral) and name of the antibiotic drug as well as updated weight. After submitting their data, patients receive a phone credit reward, irrespective of whether they have taken antibiotics or not, so that unnecessary antibiotic consumption is not encouraged. The *Tiny Guardian* prototype is available online [11].

The *Tiny Guardian’s* USSD interface offers user-friendly, text-based communication that is easy for caregivers and healthcare providers to use and requires minimal technical skills. The app encourages using and updating the child’s antibiotic history through phone credits with a control mechanism via healthcare professionals. It ensures robust security with unique IDs and PINs, safeguarding sensitive information through stringent user validation.

By integrating the USSD technology, the *Tiny Guardian* aligns with the vision of universal access to essential healthcare tools and data, thus extending its reach to communities traditionally underserved in digital health. The USSD’s potential to bridge gaps in healthcare accessibility between individuals, healthcare providers, and stakeholders, such as collecting accurate and comprehensive antibiotic usage data to curb antimicrobial resistance in children, even in the most remote and resource-limited areas, can be seen as a foundation block for other health domains like developmental follow up or vaccination in need of informed data collection, decision-making and policy formulation.

## Further Steps

We have developed the prototype of the *Tiny Guardian*, which is now ready to be put into practice by partnering with stakeholders for development, deployment, improvement, and sustainable support. Country-specific legal compliance and funding need to be secured. However, the simplicity of the USSD tool promises to be less expensive than other data collection strategies and tools. Successful testing will pave the way for the widespread deployment of *Tiny Guardian,* taking us a step further in the fight against antimicrobial resistance, promoting patient empowerment and improving child health.
